# A stable iterative method for refining discriminative gene clusters

**DOI:** 10.1186/1471-2164-9-S2-S18

**Published:** 2008-09-16

**Authors:** Min Xu, Mengxia Zhu, Louxin Zhang

**Affiliations:** 1 Program in Molecular and Computational Biology, University of Southern California, Los Angeles, CA, USA; 2 Department of Computer Science, Southern Illinois University, Carbondale, IL 62901, USA; 3 Department of Mathematics, National University of Singapore, 2 Science Drive 2, 117543, Singapore

## Abstract

**Background:**

Microarray technology is often used to identify the genes that are differentially expressed between two biological conditions. On the other hand, since microarray datasets contain a small number of samples and a large number of genes, it is usually desirable to identify small gene subsets with distinct pattern between sample classes. Such gene subsets are highly discriminative in phenotype classification because of their tightly coupling features. Unfortunately, such identified classifiers usually tend to have poor generalization properties on the test samples due to overfitting problem.

**Results:**

We propose a novel approach combining both supervised learning with unsupervised learning techniques to generate increasingly discriminative gene clusters in an iterative manner. Our experiments on both simulated and real datasets show that our method can produce a series of robust gene clusters with good classification performance compared with existing approaches.

**Conclusion:**

This backward approach for refining a series of highly discriminative gene clusters for classification purpose proves to be very consistent and stable when applied to various types of training samples.

## Background

Microarray has become an important tool for identifying genes that discriminate sample classes because of its power of monitoring the expression levels of thousands of genes in a single experiment. Finding discriminative genes with microarray data is actually the feature selection problem in classification theory. From the machine learning point of view, it is critical since the microarray datasets usually contain a small number of experiments (called samples) and a large number of genes (called features) in each experiment. The selected highly discriminative genes after filtering out those non-representative genes which may dilute the pattern in classification computation can be further studied for the investigation on the biological mechanisms that are responsible for class distinction.

A number of efforts have been put in searching effective gene selection methods (For example [1-6]). Due to the small-sample size and high-dimension properties of the tissue classification problem, it is not difficult to find a small feature subset that can perfectly discriminate all the samples [7]. In fact, theoretical study in [8] showed that even for the non-informative, randomly generated dataset, the expected size of a feature subset that can linearly discriminate all the *n *samples is just (*n *+ 1)/2. In microarray data analysis, there can be a large number of highly discriminative subsets containing only a couple of genes; and each individual gene in such a subset is not necessarily highly discriminative. For example, we observed by exhaustive search that there are as many as 10,173 perfect 3-gene subsets for classification with the weighted voting method proposed by Golub et al and with their proposed training-test split [1]; and these gene subsets cover 3,337 genes (93.4% of all the 3,571 genes in the datasets after preprocessing). This observation suggests that a method of finding a highly discriminative compact gene subset is not enough. The variability of the subsets found by such a method likely hinders the discovery of real interaction among the genes given that the method is usually sensitive to both the choice of samples and noise in the microarray data.

The fundamental limit and challenges mentioned above motivates us to design more robust methods by taking into account the expression similarity information among genes. In this paper, we identify a series of discriminative gene clusters by running clustering and feature selection processes iteratively, where the centroids of the clusters are used to form predictors. This work also shows that the predictor constructed in this way is more stable and less sensitive to the choice of training samples. Because biological functions are usually resulted from collective behavior and coordinated expression of a group of genes rather than that of an individual gene, genes grouped according to their co-expression pattern may be more powerful in revealing gene regulation mechanisms.

Our approach to generate discriminative gene clusters is a combination of supervised and unsupervised technique In recent years, Jornsten and Yu [9] and Dettling and Buhlmann [10] proposed similar combination approaches. However, there are major differences between their methods and our method. We use a multivariate approach for cluster selection, while Dettling and Buhlmann [10] employed a univariate approach, which assumes the independence of the contribution of clusters to classification. Although such hypothesis reduces computational complexity for large datasets, the accuracy is compromised since the complex biological interaction among gene clusters is not properly reflected. We exploit a multivariate approach in the content of gene expression analysis since it accounts for the joint contribution of clusters to classification.

Our method differs from [9] in the following two aspects: Although both works adopt multivariate approach, first of all, in their information-based approach, clustering and cluster selection are done simultaneously, resulting in a set of clusters optimizing the Minimum Description Length. In comparison, our computation-oriented approach is a refining process where clustering and cluster selection are performed alternatively in each iteration step with better and better results. Secondly, the clusters generated with Jornsten and Yu's approach include both active and inactive ones. Here, active clusters are those whose centroids are relevant to classification, and inactive ones are not. Our method is essentially a backward approach [4]. It iteratively eliminates the less active clusters and re-clusters the remaining genes in the active clusters, reducing the negative influence of non-discriminative clusters on the classification.

Our program outputs a series of cluster sets that have increasing discrimination power for training samples without losing prediction power on the test samples, as indicated in our experimental results. It achieved similar or better prediction accuracy than the known methods aforementioned for most of the tested datasets in our validation process. More importantly, our test shows that the centroids of the output clusters using different sets of training samples are stable and consistently achieve significant proximity to the global optimal gene clusters obtained by using all the samples. Another advantage of our method is that it provides researchers with flexibility to decide which cluster set should be chosen for their purpose.

## Results

We implemented the algorithm (described in the Methods section) as MATLAB functions. It runs on a PC with the Windows operating system. The SVM program written by Gavin Cawley was downloaded from the website . In this section, we present the detailed test results on both simulated and Leukemia AML/ALL datasets [1]. We also have tested our method on other real datasets and compared the performance of our algorithm with those reported in the previous literature. The details of the performance measures are described in the Method section.

### Simulated datasets

We generated 100 simulated binary classification datasets using a simple stochastic model. Each simulated dataset contains 100 samples evenly split into two classes. Both training and test samples contain 25 samples in each class.

Each dataset contains of 400 genes evenly divided into four gene clusters. Two of the four clusters are relevant to classification and these two discriminative clusters *C*_1 _and *C*_2 _contribute to classification independently. Their centroids *x*(*C*_1_) and *x*(*C*_2_) are generated according to the sample class labels. Each component of *x*(*C*_1_) in a position is generated according to normal distributions N(1, 0.5) or N(-1, 0.5) depending on whether the corresponding sample is in class 1 or class -1, while each component of *x*(*C*_2_) generated according to N(-1, 0.5) if the sample is in class 1 and N(1, 0.5) otherwise. Similarly, the centroids of the non-discriminative clusters *C*_3 _and *C*_4 _are generated according to the normal distribution N(1, 1) and N(-1,1) regardless of the samples' class. For each *i *= 1, 2, 3, 4, the expression values of a gene in the cluster *C*_*i *_are generated according to the multivariate normal distribution $N(x(C_{i}),\frac{d_{i}}{4})$, where *d*_*i *_= min_*j *≠ *i*_*d*(*x*(*C*_*i*_), *x*(*C*_*j*_)).

**Figure 1:**
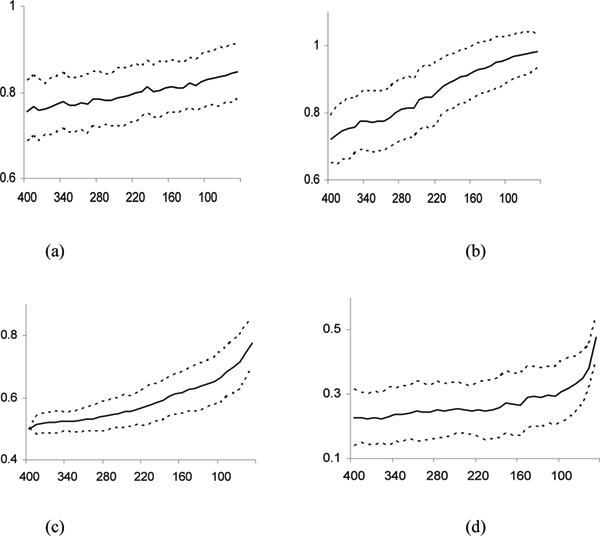
**The performance analysis on simulated datasets**. The solid lines indicate the average values; and dotted lines indicate one standard deviation from the averages. The X-axis represents the number of genes in *S*_*i*_. Note that, when the generating process goes, the number of genes in *S*_*i *_decreases. (a) The classification accuracy *θ*_*test *_on the test samples. (b) The classification accuracy *θ*_*train *_on the training samples. (c) The percentage *p*_*sim *_(*i*) of truly discriminative genes in *S*_*i*_; (d) The p-value *ρ*_*S*_(*i*) based on $\hat{\delta }$(*A*_*i*_).

We run our algorithm with the input gene set *S *contains all the 400 genes for each of the 100 simulated datasets. The performance results are summarized in Fig. 1. We observed that the classification performance of the generated clusters keeps increasing as the iteration process goes. The average classification accuracy *θ*_*test *_of these tests jumps from 0.756 up to 0.848 (Fig. 1a); and the classification accuracy *θ*_*train *_on training samples goes up from 0.720 to 0.984 (Fig. 1b).

We also observed that more and more truly discriminative genes are identified in the active clusters as the algorithm proceeds. Since the genes in the discriminative clusters are known in each simulated dataset, we computed the ratio $p_{sim}(i)=\frac{\left|S_{i}\cap (C_{1}\cup C_{2})\right|}{\left|S_{i}\right|}$ of the truly discriminative genes over all the genes in *S*_*i *_for each iteration *i*. The active clusters output *p*_*sim *_(*i*), just before the algorithm terminates is about 0.778 (Fig. 1c). Recall that, at each iteration *i*, the algorithm generates *κ *= 50 active gene clusters since the number of training samples *n*_*r *_= 50 for each simulated dataset. We found that at each iteration *i*, the centroids of two active clusters are very close to *x*(*C*_1_) and *x*(*C*_2_), the centriods of the discriminative clusters in the model. This is reflected by the indistinguishably small p-value *ρ*_*S *_(*i*) calculated based on $\bar{d}$(*A*_*i*_, Δ'). Here $\bar{d}$(*A*_*i*_, Δ') is the 'average' Euclidean distance of centroids between an active cluster in *A*_*i *_and its closest cluster in Δ' = {*C*_1_, *C*_2_}.

In the same time, the centroids of active clusters become more and more distinguishable from each other, increasingly close to the average pairwise distance of all 400 genes, and such trend can also be reflected by the increasing p-value *ρ*_*S *_(*i*) from 0.228 up to 0.476 (Fig. 1d), calculated based on $\hat{\delta }$(*A*_*i*_), the average Euclidean distance between the centroids of active clusters in *A*_*i*_. Meanwhile, the Silhouette width $\bar{\omega }$(*A*_*i*_) of active clusters in *A*_*i *_increases from 0.826 to 0.980.

### Leukemia dataset

Leukemia AML/ALL dataset [1] contains the expression values of 6,817 human genes in 47 acute lymphoblastic leukemia (ALL) and 25 acute myeloid leukemia (AML) tissue samples. After performing the threshold filtering and logarithmic transformation procedure, we obtained a reduced dataset with only 3,571 genes.

Here, we validate our algorithm by using three-fold cross validation. In each run, we randomly selected two third of the samples as the training samples and the rest as the testing samples. The samples of different classes are kept proportional in the training and test samples. The resulting dataset was further normalized by rescaling the variance of expression values of each gene to 1 in the training samples, and then applying the same rescaling factor to the expression values of that gene in the test samples.

We conducted the three-fold cross validation for 100 times. To reduce computational cost, we restrict our algorithm on small portions of discriminative genes. In each run, the algorithm starts with the input gene set *S *consisting of the 357 genes (10% of all the 3,571 genes) that are highly correlated with the training samples' classification in terms of the correlation metric proposed in [1].

**Figure 2:**
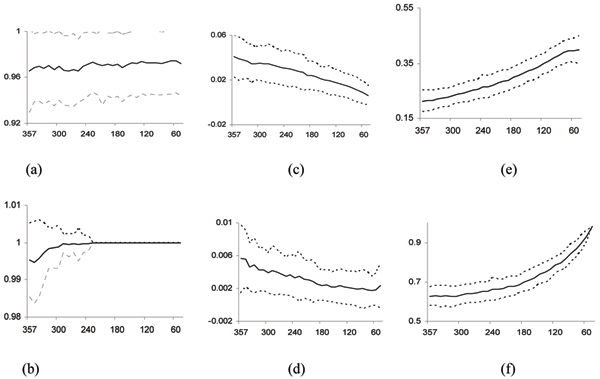
**The analysis of the three-fold cross validation performance of the algorithm on the Leukemia dataset**. The dotted lines indicate the performance values in individual tests. The solid lines indicate the average values; and the dotted lines indicate one standard deviation from the averages. The X-axis represents the number of genes in *S*_*i*_. (a) The classification accuracy *θ*_*test *_on the test samples. (b) The classification accuracy *θ*_*train *_on the training samples. (c) The p-values *ρ*_*S*_(*i*) based on $\bar{d}(A_{i},{\Delta^{\prime }}_{1})$. (d) The p-values *ρ*_*S*_(*i*) based on $\bar{d}(A_{i},{\Delta^{\prime }}_{2})$. (e) The p-value *ρ*_*all*_(*i*) based on $\hat{\delta }$(*A*_*i*_). (f) The average Silhouette width $\bar{\omega }$(*A*_*i*_) of active clusters in *A*_*i*_.

Fig. 2 summarizes the values of the different performance indicators. The average classification accuracy *θ*_*train *_on the training samples ranges from 0.994 up to 1 (Fig. 2b); and the average classification accuracy *θ*_*test *_on the test samples increase slightly from 0.966 to 0.972 (Fig. 2a). These results show that the centroids of the clusters generated in different iteration steps discriminate the training samples better and better without significant decrease of its generalization ability.

For the evaluation of our algorithm, we searched for perfect 3-gene subsets, which can be used to perfectly classify all 72 samples using the weighted voting classifier trained on all the samples. This search resulted in 9,722 perfect subsets. We selected 48 (roughly equal to *n*_*r*_) genes *g*_*i *_(1 ≤ *i *≤ 48) with highest occurrence frequency to form the cluster set ${\Delta^{\prime }}_{1}$ = {{*g*_*i*_}|1 ≤ *i *≤ 48} for comparison with the clusters generated by our algorithm.

We also evaluate our algorithm using another cluster set ${\Delta^{\prime }}_{2}$, the final set of active clusters generated by our algorithm with *S' *as the input gene subset and with all the 72 samples as the training samples, where *S' *is the set of the 357 genes (10% of all the 3,571 genes) that are highly correlated with the AML/ALL classes in terms of the correlation metric proposed in [1].

Probably because of the selection sensitivity of the correlation metric of [1] resulting from small sample size, the gene sets that are selected according to different training-test splits do not have many genes in common. In all the 100 validation experiments, only 120 genes appearing in every input gene set *S*. This number is quite small compared with 1,071, the number of the genes appearing in some input gene sets (each of size 357). By contrast, the centroids of clusters in the set *A*_*i *_generated in each of iterations of our algorithm in different runs are significantly similar to the selected discriminative genes in ${\Delta^{\prime }}_{1}$ and ${\Delta^{\prime }}_{2}$ at most iteration steps. This is reflected by the very small p-values *ρ*_*S*_(*i*) computed based on $\bar{d}(A_{i},{\Delta^{\prime }}_{1})$ and $\bar{d}(A_{i},{\Delta^{\prime }}_{2})$, which range from 4.11 × 10^-2 ^to 6.12 × 10^-3 ^(Fig. 2c) and from 5.62 × 10^-3 ^to 2.38 × 10^-3 ^(Fig. 2d) respectively. The above observation strongly suggests the stability associated with discriminative clusters rather than with individual discriminative genes. Such stability is one of the main advantages of our method.

**Figure 3:**
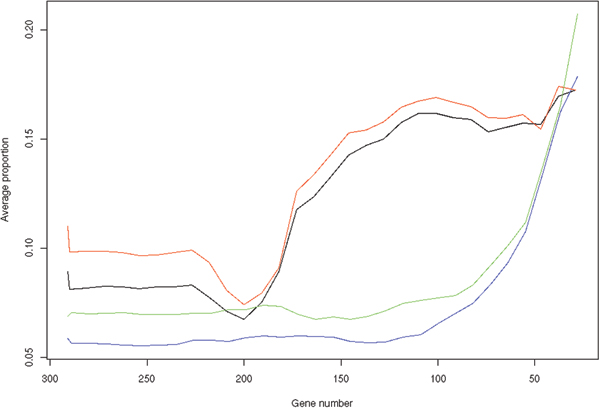
**Average proportion of genes of the four biological processes during the refinement iterations**. The solid black, blue, red, and green lines corresponding to the ordered processes in Table 1 from top to bottom.

We further studied the biological function of genes in the active clusters using Gene Ontology (GO), focusing on the biological processes located at the fifth level of the GO hierarchy. For the set of all genes from active clusters in *A*_*i*_, we find its enriched biological processes by calculating the hyper-geometric p-value, then convert the p-value into a log score *s *by *s *= -log_10 _(*p*). Table 1 gives the top four biological processes that are most significantly enriched in the active clusters in the final iteration, in terms of the score averaged from the 100 validation experiments. All four processes are frequently associated with leukemia. In addition, we inspected the change of proportion of the genes of the four processes in the active clusters during refinement iterations. The proportions are also averaged over the 100 validation experiments. Fig 3 shows that when the active clusters contain less than two third genes in input gene set *S*, the average gene proportions of all four processes monotonically increase until the last iteration. Such convergence strongly suggests that our method can indeed refine clusters into biologically meaningful ones. Interestingly, processes of inflammatory response and response to wounding showed very similar convergence patterns. In fact, these two processes are closely related. The same holds for biological processes of regulation of catalytic activity and positive regulation of metabolic process.

**Table 1:** Significantly enriched biological processes

Biological process	Average score
Inflammatory response	2.72
Regulation of catalytic activity	2.19
Response to wounding	1.98
Positive regulation of metabolic process	1.95

### The performance analysis on other real datasets

Besides the above dataset, we also tested our algorithm on seven other datasets. The descriptions of these datasets are listed in Additional file 1. Altogether, we derive 12 classification studies from the 8 datasets.

We preprocessed each dataset by applying filtering and logarithm transformation if necessary. For each classification study, we run our algorithm 100 times by choosing random training-test splits in the same way as the Leukemia dataset described in the last subsection. The performance of our method is summarized in Table 2. In the table, there are two columns for each performance measure, indicating the average values of the corresponding measures at the first and last iteration step of our algorithm. Because the exhaustive search of the most frequent globally optimal genes for constructing ${\Delta^{\prime }}_{1}$ is time-consuming, we only compare the active clusters with ${\Delta^{\prime }}_{2}$ constructed as follows: 1) we apply our algorithm on all samples in the dataset and 2) use the active clusters of the last iteration as ${\Delta^{\prime }}_{2}$.

**Table 2:** The performance of the algorithm for different classification studies

Datasets	*θ*_*test*_		*ρ*_*S*_(*i*) based on $\bar{d}(A_{i},{\Delta^{\prime }}_{2})$		*ρ*_*all*_(*i*) based on $\hat{\delta }$(*A*_*i*_)		$\bar{\omega }$(*A*_*i*_)	
Leukemia ALL T/B cell	**0.970**	**0.977**	**1.72E-02**	**8.28E-03**	0.088	0.331	0.406	0.973
Breast	0.843	0.842	**1.33E-02**	**8.36E-03**	0.142	0.421	0.351	0.974
Carcinoma	0.983	0.981	2.96E-02	3.20E-02	0.194	0.252	0.382	0.966
Colon	0.814	0.806	**2.43E-02**	**2.06E-02**	0.750	0.755	0.673	0.978
DLBCL	**0.896**	**0.929**	**8.75E-02**	**1.99E-02**	0.441	0.514	0.716	0.982
Melanoma	**0.913**	**0.921**	1.71E-02	2.25E-02	0.129	0.463	0.272	0.957
Prostate	**0.889**	**0.916**	**4.79E-02**	**2.27E-02**	0.495	0.541	0.680	0.987
SRBCT-BL	**1.000**	**1.000**	**3.63E-04**	**7.52E-05**	0.314	0.322	0.682	0.984
SRBCT-EWS	**0.956**	**0.986**	**5.06E-04**	**9.17E-05**	0.297	0.408	0.634	0.984
SRBCT-NB	**0.989**	**0.996**	**2.99E-04**	**6.42E-05**	0.321	0.436	0.665	0.986
SRBCT-RMS	**0.974**	**0.980**	**4.82E-04**	**8.18E-05**	0.304	0.347	0.630	0.989
Lukemia AML/ALL	**0.966**	**0.972**	**5.62E-03**	**2.38E-03**	0.212	0.398	0.627	0.980

The classification accuracy *θ*_*test *_on the test samples shows that among 9 of 12 classification studies, the prediction performance of active clusters in *A*_*i *_increases slightly from the start to the end of each execution, which are highlighted in the table. The value of *θ*_*test *_for the remaining three studies (Breast, Colon and Carcinoma) decrease slightly. The above observations indicate that for all datasets we tested, there is no significant decrease in the generalization ability of the active clusters in *A*_*i *_obtained in each iteration step. The classification performance *θ*_*train *_on the training samples increases in all of the 12 studies, which indicates that the separation of the training samples improves for all studies.

All the 100 input gene sets *S *vary a lot in different runs for each study. There are only 1.1% to 5.1% of all the genes appearing in all the 100 input gene sets *S*, while at least 23.8% to 51.7% genes appear in some input gene sets. By contrast, the centroids of clusters in *A*_*i *_generated by our algorithm at each iteration step *i *are stably close to the optimal centroids of clusters in ${\Delta^{\prime }}_{2}$ as reflected by the p-values *ρ*_*S*_(*i*) ranging from 2.99 × 10^-4 ^to 8.75 × 10^-2 ^at the first iteration step and those ranging from 6.42 × 10^-5 ^to 3.20 × 10^-2 ^in the last iteration step. The consistent closeness of the clusters generated in different repeats can also be reflected in the standard deviation of *ρ*_*S*_(*i*), which are limited from 0.32 to 0.96 times of the absolute values of *ρ*_*S*_(*i*) in the first iteration step and 0.24 to 1.37 times at the last iteration step.

During the generation process, the p-values *ρ*_*all*_(*i*) of average pairwise distance $\hat{\delta }$(*A*_*i*_) among centroids of clusters in *A*_*i *_keeps increasing for all 12 studies (ranging from 0.088 to 0.750 at the first iteration step and from 0.252 to 0.755 in the last step), and the average Silhouette width of active clusters $\bar{\omega }$(*A*_*i*_) keeps increasing for all the 12 studies (ranging from 0.230 to 0.698 at the first iteration step and from 0.964 to 0.989 in the last iteration step). This indicates that the clusters in *A*_*i *_are more and more distinct in general.

In summary, our test shows that on real microarray datasets, our algorithm is able to generate clusters that separate the training samples with increasing prediction accuracy and closeness to known optimal clusters. Such discriminative cluster refinement is consistent with what we have observed on simulated datasets.

### Comparing the classification performance to other studies

In this section, we compare the cross validation performance of our method with previous works reported in [9,10,15,16]. For the purpose of comparison, we converted the classification performance from the classification accuracy *θ*_*test *_into the error rate. Table 3 summarizes the comparison of our algorithm (of both binary and multi-class versions) with others by the cross validation error rates. It is difficult to make direct comparisons with other approaches in the literature, because the specific data sets or data preparation are not always available. However, the performances our method is in general comparable to others. In the comparison, the DLBCL and Carcinoma datasets are validated using leave-one-out validation; and the remaining datasets are validated using three-fold cross validation.

**Table 3:** Comparison of our algorithm with others

Datasets	Our algorithm	Dettling and Buhlmann (2002)	Jornsten and Yu (2003)	Shipp *et al. *(2002)
Lukemia AML/ALL	3.43 – 2.57	6.58 – 2.71		
Leukemia three classes	13.8 – 9.3		12.6	
Breast	16.14 – 14.11	3.00 – 0.75		
Carcinoma	5.6 – 0.0			
Colon	19.41 – 18.23	23.35 – 15.95	13.6	
DLBCL	8.7 – 7.4			7.8
Prostate	11.09 – 8.36	16.47 – 6.91		
SRBCT multi class	5.92 – 4.27	5.76 – 0.43		

Dettling and Buhlmann [10] reported the error rate of their algorithm for different datasets. They employed nearest neighbors and aggregated trees as the classifiers in their three-fold cross validation test. For the leukemia AML/ALL dataset, our algorithm seems to achieve a slightly lower error rate than theirs. In the Colon and Prostate datasets, the error rate of our algorithm lies between that of theirs. For the Breast dataset, the error rate is significantly higher than that of Dettling and Buhlmann's. However, we obtained the performance using all the original 49 samples. The error rate in each test ranges from 7.89 to 6.90. According to [17], at least 7 out of the 49 samples are inherently erroneous. In comparison, Dettling and Buhlmann [10] used the 38 good samples selected by [17], and the error rate ranges from 1.14 to 0.10. The 38 samples used in Dettling and Buhlmann [10] consists none of the above 7 erroneous samples. Thus, we believe that the performances of ours and Dettling and Buhlmann's are still comparable for the Breast dataset.

For the DLBCL dataset, the leave-one-out performance of Shipp et al. [15] is in our performance range. For Carcinoma dataset, Jaeger et al. [16] achieved perfect leave-one-out performance, and our best performance can match theirs. For the Colon dataset, both ours and Dettling and Buhlmann's error rate are higher than Jornsten and Yu's.

We also test the performance of the multiple-class version of our method against other methods. For the Leukemia three-class dataset, our method is comparable to Jornsten and Yu's method. However, for the SRBCT multi class dataset, our algorithm seems had a slightly higher error rate than that of Dettling and Buhlmann's.

## Conclusion

Due to the small-sample-high-dimension nature of the microarray dataset, it is not difficult to find highly discriminative gene subsets of small size. However, if a gene selection process is unstable with the choice of training samples, the biological significance of the resulting gene subsets is often not guaranteed. In this paper, instead of finding individual discriminative genes or gene subsets, we propose a novel backward approach for generating a series of highly discriminative gene clusters. Compared to selection of individual discriminative genes, genes grouped in these clusters are more stable when subject to change of training samples. Therefore they could provide more convincing support to gene interactions that are associated with the sample classes. In future, we will work with biologists to study the biomedical implication of these clusters.

Regarding to the classification performance, the gene clusters produced by our approach can generally achieve good cross validation performance compared to the existing methods for most of datasets we tested. More importantly, our test experiments show that regardless of the choice of training samples, the centroids of the clusters generated are stable and significantly close to the known optimal gene clusters found using all the samples. All these indicate that our approach is promising. However, the current version of our algorithm is time-consuming. In future, the computational efficiency will be investigated. On the other hand, we used K-means algorithm, a typical partitioning based clustering method to seek a certain number of clusters that minimize the sum of squared distances between each gene and its centroid. The drawback for K-means is the subjective specification of input parameters such as the number of clusters and initial centroid locations. For unknown microarray datasets, such information is unavailable. Furthermore, different input parameters may result in significantly different clustering results. K-means can only converge to local optima, rather than the global optimum. In order to address these problems associated with K-means clustering. We plan to apply a novel clustering method based on Random Matrix Theory (RMT) [18] which is completely objective and do not require the specification of cluster number and initial centroid locations. RMT method avoids being trapped into local optima. Furthermore, most previous clustering methods including K-means and hierarchical clustering partition members into non-overlapping groups. The RMT method allows the same genes in multiple groups to reflect the fact that a single gene may contribute to multiple biological pathways.

In order to test the discriminative power of a certain gene cluster, additional criteria established by statistical analysis should also be conducted to identify and remove inactive cluster. For example, gene expression pattern observed in the active clusters should be less likely to appear in the control set. Chi Square test might be used to test the significance. Some data normalization technique may be considered in the preprocessing step to improve the data quality. Furthermore, more suitable backward feature selection method needs to be exploited so that the gene clustering and cluster selection processes can be integrated better. Our approach provides a flexible framework that allows us to test the performance of various computing modules in a various ways of combinations.

## Methods

### Algorithm

In this subsection, we present our backward approach for generating discriminative gene clusters. The method is executed in a repetitive manner. In each pass, the method first groups genes into clusters that may indicate functional categories [11]. It then ranks the generated clusters and eliminates those clusters that are less discriminative so that the re-clustering of remaining genes can generate modules with better resolution and stronger association with the sample classes. In the clustering stage, we use the K-means method to group the genes into a constant number of active clusters.

In the elimination stage, we use a backward feature selection method. This stage involves cluster validation and evaluation of the discriminative ability of active clusters. To validate clusters, we use the Silhouette width [12] to measure their validity. Assume the input genes are partitioned into *p *clusters *C*_1_, *C*_2_,..., *C*_*p*_. Given a gene *g*, let ${\bar{w}}_{g}$ be the average Euclidian distance between *g *and another gene within the same cluster, and let ${\bar{b}}_{gJ}$ the average Euclidian distance between *g *and a gene in a different cluster *C*_*J*_. Then the Silhouette width *w*_*g *_of *g *is defined as $\omega_{g}=\frac{\underset{J}{\min}\left({\bar{b}}_{gJ}\right)-{\bar{w}}_{g}}{\max\left(\underset{J}{\min}\left({\bar{b}}_{gJ}\right),{\bar{w}}_{g}\right)}$, and the Silhouette width of a cluster is defined as the average Silhouette width of all its members. It is easy to see that the Silhouette width of a cluster fall within the range from -1 to 1. A good cluster should have a high Silhouette width.

To measure the discriminative ability of an active cluster, we adopt the idea of SVM-RFE method in [2]. Support Vector Machine (SVM) is a binary-class prediction method originated from statistical learning theory [13]. A linear SVM first finds a decision hyperplane *y *= **w**^T^**x **+ *b *that maximizes the separation between samples of two classes; and then it does class prediction according to the relative location of a new sample with respect to the hyperplane in the feature space. Note that the weight vector **w **found by the linear SVM indicates the relative importance of the genes for the classification. Here, we iteratively train a linear SVM and eliminate a gene cluster based on an overall evaluation on both the weight and the Silhouette width instead of discarding single gene in the original SVM-RFE method. Such systematic approach makes the elimination process to better reflect the underlying biological meaning.

**Figure 4:**
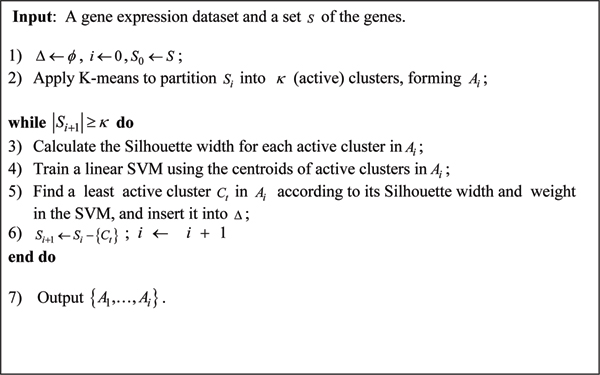
The algorithm of selecting discriminative clusters.

Our method is summarized into the algorithm in Fig. 4. In the algorithm, Δ denotes the set of inactive gene clusters; *A*_*i *_denotes the set of active clusters at each iteration *i*; *S*_*i *_denotes the set of genes under consideration at the beginning of the iteration *i*; *κ *denotes the number of clusters partitioned at each iteration step. For simplicity, we set *κ *to be *n*_*r*_, the number of training samples.

It is often difficult to determine how many clusters the genes should be grouped into for microarray datasets, which usually have complex expression patterns. The algorithm outputs *κ *= *n*_*r*_, the number of the training samples, active clusters in each iteration. This is because the expected size of a feature subset that can linearly discriminate all the samples is only (*n*_*γ *_+ 1)/2 [8]. Note that if the feature number is too small, the clustering will lose its resolution.

Recall that the K-means clustering method starts with an initial partition of the genes. In order to make it more deterministic in Step 2, we first select *κ *genes as follows: Find a furthest gene pair and form an initial gene set G, and then iteratively find a gene with largest average Euclidean distance from the genes in G and add it into G until |*G| *= *κ*. We then partition all the genes into *κ *clusters by merging each gene with its nearest gene in G.

The calculation of the Silhouette width of each cluster in *A*_*i *_takes all the clusters in both sets *A*_*i *_and Δ into account. At the *i*th iteration, the algorithm groups all the genes in the set *S*_*i *_into *κ *clusters, forming the cluster set *A*_*i*_, and then insert the least active cluster into the inactive cluster set Δ in Step 5 as follows.

There are two important factors to evaluate in order to determine which cluster should be removed from *S*_*i *_and added into Δ. The first factor is the cluster's Silhouette width. Another factor is the cluster's discriminative ability in terms of its weight determined by the linear SVM constructed in Step 4. Here, we would like to eliminate a least discriminative cluster whose centroid is sufficiently representative of the expression pattern of the cluster (measured by the Silhouette width). In other words, we eliminate a set of well clustered genes whose expression patterns have little contribution to classification. On the other hand, those not well clustered genes will be re-clustered at later iterations.

**Figure 5:**
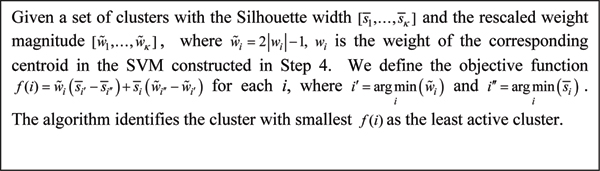
Multiple objective optimization procedure for cluster elimination.

Since the above two factors are not always consistent, we adopt a multiple objective optimization technique appearing in [14] to find a nice tradeoff between these two factor and such multiple objective method is shown in Fig. 5.

Finally, we can extend the algorithm to the multiple-class case by adopting the popular one-against-all approach. In this approach, given a training test split, both training and test samples of a dataset of *K *> 2 classes are transferred into *K *binary classification problems, each corresponding to classify samples from one class against samples from all remaining classes. Then our algorithm executed on the *K *problems results in *K *series of active cluster sets *A*_*j*, *i*_, *j *= 1,..., *K*. Then classifiers are constructed using *K *× *κ *clusters from the *K *active cluster sets $A_{1,i_{1}},...,A_{k,i_{K}}$ by selecting *i*_1_,..., *i*_*K *_such that |$S_{i_{1}}$|,..., |$S_{i_{K}}$| are roughly identical. Given the centroids of the above *K *× *κ *clusters, a multi-class linear SVM is trained using training samples and tested on test samples.

### Performance measures

We validate our method in terms of its classification performance and clustering performance. The classification performance is determined by the classification accuracy on training or testing samples. We use the SVM as the classifier to evaluate the generated gene clusters. Classification accuracy *θ*_*test *_on the test samples is defined as the percentage of the correctly classified samples. However, we define classification accuracy *θ*_*train *_on training samples as the average accuracy of the 10-fold cross validation on the training samples as suggested in [4] for less biased estimation of classification performance.

The clustering quality is measured in terms of the density of the clusters, as well as the distinction between clusters and the closeness of the clusters to some reference clusters. They are measured respectively by (a) the average Silhouette width $\bar{\omega }$(*A*_*i*_) of active clusters in *A*_*i *_produced in iteration *i*, (b) the average Euclidean distance $\hat{\delta }$(*A*_*i*_) between the centroids of active clusters in *A*_*i *_and (c) the 'average' Euclidean distance $\bar{d}$(*A*_*i*_, Δ') of centroids between the an active cluster in *A*_*i *_and its closest cluster in a reference cluster set Δ' (the construction can be found in the Result Section). To be more precise, assume *A*_*i *_= {*C*_1_,..., *C*_*κ*_} and Δ' = {*D*_1_,..., *D*_*κ*_}. First, from 1 to *κ*, find recursively ${C^{\prime }}_{l}$ ∈ *A*_*i *_and ${D^{\prime }}_{l}$ ∈ Δ' such that $d(x({C^{\prime }}_{l}),x({D^{\prime }}_{l}))=\min_{{C^{\prime }}_{l}\in A_{i}-A^{\prime \prime },{D^{\prime }}_{l}\in \Delta^{\prime }-{\Delta^{\prime \prime }}_{l}}d(x({C^{\prime }}_{l}),x({D^{\prime }}_{l}))$, where *x*() denotes the centeroid of a cluster, *d*(,) the Euclidean distance between two vectors, $A^{\prime \prime }=\{{C^{\prime }}_{1},...,{C^{\prime }}_{l-1}\}$ and ${\Delta^{\prime \prime }}_{l}=\{{D^{\prime }}_{1},...,{D^{\prime }}_{l-1}\}$. Then, the 'average' Euclidean distance $\bar{d}$(*A*_*i*_, Δ') is defined as $\bar{d}(A_{i},\Delta^{\prime })=\frac{1}{\kappa }\sum_{1\leq l\leq \kappa }d(x({C^{\prime }}_{l}),x({D^{\prime }}_{l}))$.

We measure the statistical significance of average distances in both case (b) and (c) at each iteration *i *against the pairwise distances of all genes in the input gene set *S *in terms of the p-value *ρ*_*S*_(*i*), and against all the genes in the dataset in terms of the p-value *ρ*_*all*_(*i*). In each case, the p-values are calculated according to the empirical distribution (null distribution) of the pairwise distance of genes randomly sampled in the whole dataset.

## Competing interests

The authors declare that they have no competing interests.

## Authors' contributions

MX carried out programming and data analysis. MX and LXZ designed the projects and drafted the manuscript. MZ participated in data analysis and improvement of the method. All authors have read and approved the final manuscript.

## Supplementary Material

Additional file 1 **Supplementary document**. This document includes description of the 8 datasets used in the result section. Click here for file
