# Supplementary material for: A stable iterative method for refining discriminative gene clusters
Source: BMC Genomics. 2008 Sep 16;9(Suppl 2):S18. doi: 10.1186/1471-2164-9-S2-S18 (PMC2559882; doi:10.1186/1471-2164-9-S2-S18)
Supplement: Additional file 1 — Supplementary document. This document includes description of the 8 datasets used in the result section. [file 1471-2164-9-S2-S18-S1.pdf]

**The performance analysis on other real datasets**

We also tested our algorithm on the following datasets:

(1) ALL T/B Cell dataset. The 47 ALL samples in Leukemia ALL/AML dataset in [1] are further divided into 39 T-cell samples and 9 B-cell samples.

(2) Breast cancer dataset [2]. This dataset comprises 7,129 genes and 49 samples being divided into two classes according to their estrogen receptor (ER) responses: 25 for ER positive and 24 for ER negative.

(3) Carcinoma dataset [3]. It contains the expression levels of about 6600 genes in 18 tumor and 18 normal tissues.

(4) Colon dataset [4]. It consists of the expression levels of 6,500 human genes in 40 tumor and 22 normal tissues.

(5) Diffuse Large B-Cell Lymphoma (DLBCL) dataset [5]. It consists of expression values of 6,817 genes in 58 DLBCL and 19 Follicular Lymphoma.

(6) The Melanoma dataset [6]. The dataset consists of the expression ratios of 6,971 human genes in 12 Unclustered Cutaneous Melanomas and 19 Cutaneous Melanomas samples.

(7) Prostate dataset [7]. The dataset consists of the expression levels of 52 prostate and 50 normal samples of 6,744 human genes.

(8) The Small, round blue cell tumors (SRBCT) dataset [8]. It consists of 88 samples divided into five classes: neuroblastoma (NB) (18 samples), rhabdomyosarcoma (RMS) (25 samples), Burkitt lymphomas (BL) (11 samples), the Ewing family of tumors (EWS) (29 samples) and others (5 samples). The dataset has 2,308 genes. Since we consider the binary classification problem, we derived four binary classification datasets from this dataset using the one-against-all rule: SRBCT-NB, SRBCT-RMS, SRBCT-BL SRBCT-EWS.

RFERENCES


1. Golub TR, Slonim DK, Tamayo P, Huard C, Gaasenbeek M, Mesirov JP, Coller H, Loh ML, Downing JR, Caligiuri MA *et al*: **Molecular classification of cancer: class discovery and class prediction by gene expression monitoring.** *Science* 1999, **286**(5439):531-537.
2. West M, Blanchette C, Dressman H, Huang E, Ishida S, Spang R, Zuzan H, Olson JA, Jr., Marks JR, Nevins JR: **Predicting the clinical status of human breast cancer by using gene expression profiles.** *Proc Natl Acad Sci U S A* 2001, **98**(20):11462-11467.
3. Notterman DA, Alon U, Sierk AJ, Levine AJ: **Transcriptional gene expression profiles of colorectal adenoma, adenocarcinoma, and normal tissue examined by oligonucleotide arrays.** *Cancer Res* 2001, **61**(7):3124-3130.

4. Alon U, Barkai N, Notterman DA, Gish K, Ybarra S, Mack D, Levine AJ: **Broad patterns of gene expression revealed by clustering analysis of tumor and normal colon tissues probed by oligonucleotide arrays.** *Proc Natl Acad Sci U S A* 1999, **96**(12):6745-6750.
5. Shipp MA, Ross KN, Tamayo P, Weng AP, Kutok JL, Aguiar RC, Gaasenbeek M, Angelo M, Reich M, Pinkus GS *et al*: **Diffuse large B-cell lymphoma outcome prediction by gene-expression profiling and supervised machine learning.** *Nat Med* 2002, **8**(1):68-74.
6. Bittner M, Meltzer P, Chen Y, Jiang Y, Seftor E, Hendrix M, Radmacher M, Simon R, Yakhini Z, Ben-Dor A *et al*: **Molecular classification of cutaneous malignant melanoma by gene expression profiling.** *Nature* 2000, **406**(6795):536-540.
7. Singh D, Febbo PG, Ross K, Jackson DG, Manola J, Ladd C, Tamayo P, Renshaw AA, D'Amico AV, Richie JP *et al*: **Gene expression correlates of clinical prostate cancer behavior.** *Cancer Cell* 2002, **1**(2):203-209.
8. Khan J, Wei JS, Ringner M, Saal LH, Ladanyi M, Westermann F, Berthold F, Schwab M, Antonescu CR, Peterson C *et al*: **Classification and diagnostic prediction of cancers using gene expression profiling and artificial neural networks.** *Nat Med* 2001, **7**(6):673-679.
9. Dettling M, Buhlmann P: **Supervised clustering of genes.** *Genome Biol* 2002, **3**(12):RESEARCH0069.
10. Jornsten R, Yu B: **Simultaneous gene clustering and subset selection for sample classification via MDL.** *Bioinformatics* 2003, **19**(9):1100-1109.
11. Jaeger J, Sengupta R, Ruzzo WL: **Improved gene selection for classification of microarrays.** *Pac Symp Biocomput* 2003:53-64.
